# Surveillance of COVID-19–Associated Multisystem Inflammatory Syndrome in Children, South Korea

**DOI:** 10.3201/eid2704.210026

**Published:** 2021-04

**Authors:** Young June Choe, Eun Hwa Choi, Jong Woon Choi, Byung Wook Eun, Lucy Youngmin Eun, Yae-Jean Kim, Yeo Hyang Kim, Young A. Kim, Yun-Kyung Kim, Ji Hee Kwak, Hyuk Min Lee, Hyunju Lee, Joon Kee Lee, June Dong Park, Eun-Jin Kim, Young Joon Park, Jin Gwack, Sang Won Lee

**Affiliations:** Korea University Anam Hospital, Seoul, South Korea (Y.J. Choe);; Seoul National University College of Medicine, Seoul (E.H. Choi, H. Lee, J.D. Park);; Bundang Jesaeng General Hospital, Seongnam, South Korea (J.W. Choi);; Eulji University School of Medicine, Seoul (B.W. Eun); Yonsei University College of Medicine, Seoul (L.Y. Eun, H.M. Lee);; Sungkyunkwan University School of Medicine, Seoul (Y.-J. Kim, J.H. Kwak);; School of Medicine Kyungpook National University, Daegu, South Korea (Y.H. Kim);; Pusan National University Children's Hospital, Yangsan, South Korea (Y.A. Kim);; Korea University College of Medicine, Seoul (Y.-K. Kim);; Chungbuk National University Hospital, Cheongju, South Korea (J.K. Lee);; Korea Disease Control and Prevention Agency, Cheongju (E.-J. Kim, Y.J. Park, J. Gwack, S.W. Lee)

**Keywords:** Coronavirus disease, COVID-19, multisystem inflammatory syndrome, pediatric, child, inflammation, surveillance, MIS-C, SARS-CoV-2, severe acute respiratory syndrome coronavirus 2, viruses, respiratory infections, zoonoses, South Korea

## Abstract

A concerning development during the coronavirus disease pandemic has been multisystem inflammatory syndrome in children. Reports of this condition in East Asia have been limited. In South Korea, 3 cases were reported to the national surveillance system for multisystem inflammatory syndrome in children. All case-patients were hospitalized and survived with no major disease sequelae.

Amid the coronavirus disease (COVID-19) pandemic, multisystem inflammatory syndrome in children (MIS-C) has emerged as a major concern globally (*1*). MIS-C features clinical characteristics that overlap with Kawasaki disease, including high fever, mucocutaneous involvement, and affecting of coronary arteries. Yet, reports of MIS-C have been limited in East Asia countries, where the incidence of Kawasaki disease is high (*2*).

Although South Korea was one of the countries struck early in the COVID-19 pandemic, spread of the virus there has been relatively contained. However, reports on MIS-C from other countries has necessitated the monitoring of COVID-19–associated MIS-C at the national level. In May 2020, the Korean Society of Pediatric Infectious Diseases, Korean Society of Kawasaki Disease, and Korean Society of Pediatric Critical Care Medicine, with support from the Korea Disease Control and Prevention Agency, created a strategic framework for prospective surveillance of MIS-C in South Korea. In this study, we describe the development of the MIS-C surveillance system and report the clinical characteristics of children meeting the case definition of MIS-C in South Korea.

## The Study

First, the Case Assessment Committee (CAC) was established, consisting of 4 pediatric infectious disease specialists, 3 pediatric cardiologists, 3 pediatric intensivists, 1 clinical microbiologist, and 1 epidemiologist. A case reporting form was created, and members of the Korean Pediatric Society (n = 5,891) were contacted to provide assistance with data collection and reporting.

Once a suspected MIS-C case was reported, CAC members quickly assessed whether the case met the clinical criteria for MIS-C case definition. In accordance with the Infectious Disease Control and Prevention Act (chapter 4, article 18), the public health officers then conducted an epidemiologic investigation of all suspected MIS-C cases. For all reported cases, the Korea Disease Control and Prevention Agency performed serologic assays for severe acute respiratory syndrome coronavirus 2 (SARS-CoV-2), including neutralizing antibody tests and the Anti-SARS-CoV-2 ELISA Assay for detection of IgG (EUROIMMUN, https://www.euroimmun.com). CAC meetings were held on an ad hoc basis for case ascertainment, treatment consultation, and exchange of knowledge. The study was approved by the Institutional Review Board of Seoul National University Hospital (approval no. 2012–136–118).

During May–November 2020, a total of 2,287 COVID-19 cases in persons 0–19 years of age were reported (Figure). During the surveillance period, 9 suspected cases of MIS-C were reported to the surveillance system. Of the reported cases, 3 (33%) case-patients had evidence of COVID-19 exposure (positive for SARS-CoV-2 by PCR, SARS-CoV-2 antibody detection, or exposure history), and their illness was assessed as COVID-19–associated MIS-C, which likely occurred 3–4 weeks after the diagnosis of COVID-19 (Table).

**Figure:**
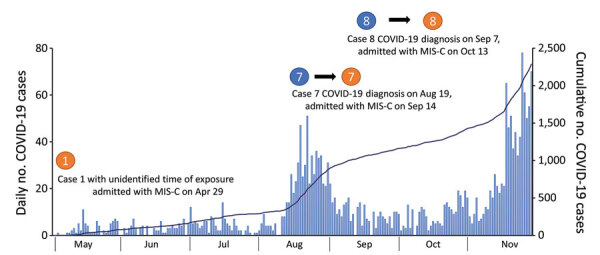
Daily number (bars) and cumulative number (line) of COVID-19 cases among children 0–19 years of age, South Korea, May–November 2020. The occurrences of the 3 cases of multisystem inflammatory syndrome are indicated. COVID-19, coronavirus disease; MIS-C, multisystem inflammatory syndrome in children.

**Table:** Demographics, clinical features, treatments, and outcomes of the 3 COVID-19–associated MIS-C case-patients, South Korea, May–November 2020*

Characteristics	Case 1	Case 2	Case 3
Age, y	11	11	14
Sex	Boy	Boy	Girl
Underlying disease	None	None	None
Clinical signs and symptoms			
Initial symptoms	Fever, abdominal pain	Fever, abdominal pain, headache, nausea, vomiting	Fever, abdominal pain, diarrhea
Fever	Present	Present	Present
Conjunctival injection	Present	Present	Present
Mucosal change	Present	None	Present
Skin rash	Present	None	Present
Extremity changes	Present	None	Present
Lymphadenopathy	None	None	None
Gastrointestinal symptoms	Present	Present	Present
Hypotension	Present	Present	Present
Inflammatory markers (peak)			
Leukocyte (neutrophil %), 10^3^/μL	7.55 (87)	9.55 (82.8)	26.56 (93)
ESR, mm/h	NT	82	77
CRP, mg/L	18.95	10.36	>30
Fibrinogen, mg/dL	633	NT	NT
Procalcitonin, ng/mL	14.55	1.54	9.62
D-dimer, μg/mL	894	2.5	3.95
Ferritin, μg/mL	NT	2485	663
IL-6, pg/mL	NT	NT	2410
Abnormal imaging studies			
Echocardiography	Coronary dilatation	Mitral regurgitation	Coronary dilatation, left ventricle dysfunction
Chest radiography or CT	Bilateral pleural effusion, pneumonic infiltration	Suspected pulmonary edema	Bilateral pulmonary edema, pleural effusion
Abdominal ultrasound or CT	Abdominal lymphadenopathy	Mesenteric lymphadenopathy	Hyperechoic liver, gallbladder hypertrophic edema, peripancreatic fluids, splenomegaly, scant pelvic ascites
Treatment			
IVIg	Provided	Provided	Provided
ASA	Provided	Provided	Provided
Steroids	Not provided	Not provided	Provided
Immunomodulatory	Not provided	Not provided	Provided (Anakinra)
Inotropic agent	Provided	Not provided	Provided
ICU care	Provided	Not provided	Provided
Mechanical ventilator	Not provided	Not provided	Not provided
Outcome			
Hospitalization, d	12 d	10 d	19 d
ICU admission, d	6 d	NA	7 d
Prognosis	Improved, discharged	Improved, discharged	Improved, discharged

*MIS-C clinical case definition is as follows: age <19 y, fever >38.0°C for >24 h, laboratory evidence of inflammation (i.e., elevation of ESR, CRP, fibrinogen, procalcitonin, d-dimer, ferritin, LDH, IL-6, neutrophilia, lymphopenia, hypoalbuminemia), multisystem involvement (>2 organ systems involved), severe illness requiring hospitalization, and no other plausible microbial cause of inflammation (i.e., bacterial sepsis, staphylococcal/streptococcal toxic shock syndromes, enteroviral myocarditis). Evidence of SARS-CoV-2 exposure history defined as positive SARS-CoV-2 by RT-PCR, positive serology (neutralizing antibody or anti-SARS-CoV-2 IgG), or exposure to individual with COVID-19 <4 weeks before onset of symptoms (epidemiologic linkage with individual or cluster). ASA, acetylsalicylic acid; COVID-19, coronavirus disease 2; CRP, c-reactive protein; CT, computed tomography; ESR, erythrocyte sedimentation rate; ICU, intensive care unit; IL-6, interleukin 6; IVIg, intravenous immunoglobulin; LDH, lactate dehydrogenase; MIS-C, multisystem inflammatory syndrome in children; NA, not applicable; NT, not tested; RT-PCR, reverse transcription PCR; SARS-CoV-2, severe acute respiratory syndrome coronavirus 2.

The age of case-patients ranged from 11 to 14 years, 2 were boys, and none had preexisting conditions. All case-patients had fever and abdominal symptoms (abdominal pain, nausea, vomiting, or diarrhea) at admission. Mucocutaneous symptoms and signs (mucosal changes, skin rash, extremity changes) occurred in 2 patients, and all patients had documented hypotension (<50th percentile, adjusted for age, sex, and height). All case-patients had marked leukocytosis or elevated inflammatory markers. Echocardiography showed coronary artery dilatation (z-scores 1.64–3.98 mm for left coronary arteries), mitral regurgitation, or left ventricular dysfunction. Chest radiography or computed tomography showed pulmonary edema or pleural effusion. Abdominal ultrasound or computed tomography showed mesenteric lymphadenopathies, hyperechoic liver, or hypertrophic gall bladder. All 3 case-patients received intravenous immunoglobulin (IVIg); 1 patient (case 3) received methylprednisolone pulse therapy and immunomodulatory agent (Anakinra) because of persistent hypotension after initial IVIg treatment. Two patients received inotropic agents and required transfer to the intensive care unit (ICU), but no patients required mechanical ventilation. The duration of hospitalization was 10–19 days, and duration of ICU admission was 6–7 days. All 3 patients received aspirin and have survived to date with no major disease sequelae.

## Conclusions

We describe MIS-C surveillance results from South Korea, an East Asia country with high incidence of Kawasaki disease. As of December 15, 2020, COVID-19 had been diagnosed in 4,107 children and adolescents 0–19 years of age in South Korea, which translates roughly to 0.07% of all childhood COVID-19 cases reported in South Korea (*3*). Concern about MIS-C was raised after episodes of increased incidence of Kawasaki-like disease were noted in children after COVID-19 diagnosis in Europe and the United States (*4*,*5*). In South Korea, there was no substantial increase in Kawasaki disease–related hospitalizations in 2020 compared with 2016–2019 (*6*). There might be ethnic differences in susceptibility; only 5% of MIS-C cases in New York (USA) occurred in Asian persons (*7*). Reports from India (*8*), Pakistan (*9*), and Iran (*10*) underscore the importance of monitoring MIS-C cases; however, surveillance data have not yet been reported for East Asia countries. Alongside genetic susceptibility, the background incidence of SARS-CoV-2 infection might play a critical role in the occurrence of MIS-C. 

Although estimates of risk for MIS-C after SARS-CoV-2 infection are not yet available, we report a rough estimate in South Korea, where COVID-19 testing is widely accessible (*11*). Our findings suggest that the incidence of MIS-C is low among children with COVID-19 in this country. However, COVID-19–associated MIS-C might cause serious clinical outcomes requiring ICU care and might require immunomodulatory agents.

All 3 MIS-C case-patients experienced gastrointestinal symptoms, which is consistent with reports from Italy (*5*), the United States (*12*), and the United Kingdom (*13*) that indicate gastrointestinal symptoms appear to be the most prominent clinical manifestation of MIS-C. Gastrointestinal involvement might also be a predictor of severe COVID-19. A systematic review of 83 studies showed that diarrhea (odds ratio 1.50, 95% CI 1.10–2.03; p = 0.01) was observed more often in patients with severe COVID-19 compared with patients with non-severe COVID-19 (*14*). Previously, syndromic involvement of the gastrointestinal system has been associated with higher risk for IVIg resistance and coronary aneurysms in patients with Kawasaki disease (*15*). These features indicate the possibility of a mechanism linking gastrointestinal involvement and syndromic features for MIS-C and Kawasaki-like illness, which needs further elucidation.

The first limitation of this study is that, given the intrinsic properties of a passive surveillance system, only a fraction of actual MIS-C cases might have been reported. Pediatricians are more likely to report cases that result in serious conditions; nonetheless, the case definition included hospitalization. Second, a large proportion of SARS-CoV-2 infections in children are asymptomatic; therefore, passive surveillance that relies on the presence of symptoms might underestimate the actual incidence of MIS-C. Despite these limitations, this study suggests that enhanced passive surveillance, including frequent outreach to pediatricians through academic societies, was a manageable scheme to monitor MIS-C in South Korea. Given that the level of SARS-CoV-2 community transmission was low during the surveillance period, passive surveillance was considered a robust plan to capture MIS-C cases at a national level.

Despite the introduction of vaccines, the global COVID-19 pandemic could continue for months. Therefore, surveillance is a critical tool for the detection and evaluation of serious complications in vulnerable population. Our experience offers a possible surveillance model for other countries concerned about COVID-19–associated MIS-C. MIS-C surveillance data in South Korea call for enhanced monitoring through syndromic and laboratory-based combination surveillance approaches.
